# 3,3′-Dimethyl-1,1′-ethyl­ene­diimidazolium dibromide

**DOI:** 10.1107/S1600536809036009

**Published:** 2009-09-12

**Authors:** Yu Chen, Wei Song, Juan Xu, Rui-bo Cui, Dan-bi Tian

**Affiliations:** a Department of Applied Chemistry, College of Science, Nanjing University of Technology, Nanjing 210009, People’s Republic of China

## Abstract

The title compound, C_10_H_16_Br_2_N_4_, was synthesized by the reaction of 1-methyl­imidazole and 1,2-dibromo­ethane in toluene. The complete dication is generated by a crystallographic inversion centre situated at the mid-point of the ethane C—C bond. In the crystal structure, weak inter­molecular C—H⋯Br inter­actions link the mol­ecules into chains along the *b* axis and an intramolecular C—H⋯Br close contact is also present.

## Related literature

For general background, see: Ding *et al.* (2007 ▶). For related literature, see: Peveling (2001 ▶); Takao & Kazuhiko (1997 ▶). For bond-length data, see: Allen *et al.* (1987 ▶). 
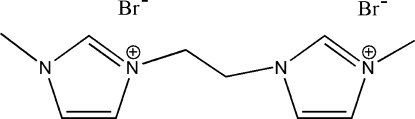


## Experimental

### 

#### Crystal data

C_10_H_16_N_4_^2+^·2Br^−^
*M* *_r_* = 352.07
Monoclinic, 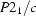

*a* = 8.4750 (17) Å
*b* = 8.9620 (18) Å
*c* = 9.2390 (18) Å
β = 107.73 (3)°
*V* = 668.4 (3) Å^3^
*Z* = 2
Mo *K*α radiation
μ = 6.05 mm^−1^
*T* = 293 K
0.30 × 0.20 × 0.10 mm

#### Data collection

Enraf–Nonius CAD-4 diffractometer
Absorption correction: ψ scan (North *et al.*, 1968 ▶) *T*_min_ = 0.264, *T*_max_ = 0.583
1296 measured reflections
1212 independent reflections
862 reflections with *I* > 2σ(*I*)
*R*_int_ = 0.021
3 standard reflections every 200 reflections intensity decay: 1%

#### Refinement

*R*[*F* ^2^ > 2σ(*F* ^2^)] = 0.059
*wR*(*F* ^2^) = 0.160
*S* = 1.01
1212 reflections
73 parameters
H-atom parameters constrained
Δρ_max_ = 0.80 e Å^−3^
Δρ_min_ = −0.85 e Å^−3^

### 

Data collection: *CAD-4 EXPRESS* (Enraf–Nonius, 1985 ▶); cell refinement: *CAD-4 EXPRESS*; data reduction: *XCAD4* (Harms & Wocadlo,1995 ▶); program(s) used to solve structure: *SHELXS97* (Sheldrick, 2008 ▶); program(s) used to refine structure: *SHELXL97* (Sheldrick, 2008 ▶); molecular graphics: *SHELXTL* (Sheldrick, 2008 ▶); software used to prepare material for publication: *SHELXTL*.

## Supplementary Material

Crystal structure: contains datablocks I, global. DOI: 10.1107/S1600536809036009/at2872sup1.cif

Structure factors: contains datablocks I. DOI: 10.1107/S1600536809036009/at2872Isup2.hkl

Additional supplementary materials:  crystallographic information; 3D view; checkCIF report

## Figures and Tables

**Table 1:** Hydrogen-bond geometry (Å, °)

*D*—H⋯*A*	*D*—H	H⋯*A*	*D*⋯*A*	*D*—H⋯*A*
C2—H2*A*⋯Br	0.93	2.92	3.591 (8)	130
C1—H1*B*⋯Br^i^	0.96	2.97	3.738 (8)	138

Symmetry code: (i) 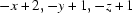.

**Fig. 1.**
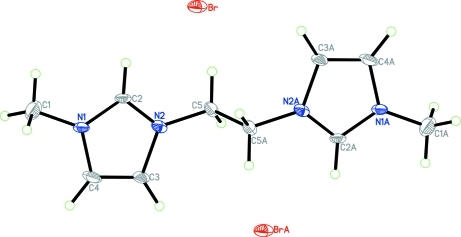
A drawing of the title molecular structure, with the atom-numbering scheme. Displacement ellipsoids are drawn at the 50% probability level. Atoms labeled with the suffixes A are generated by the symmetry operation ( -x+1, -y+1, -z+1). Hydrogen bonds are shown by dashed lines.

**Fig. 2.**
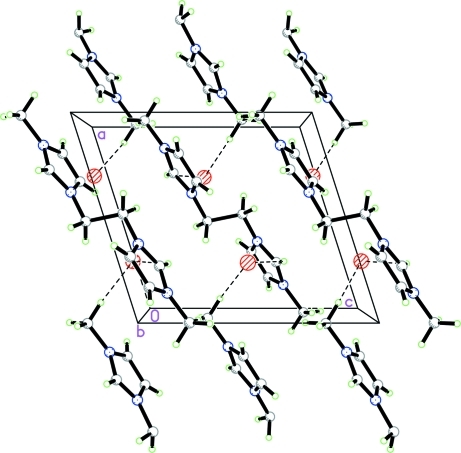
A packing diagram for (I).

## supplementary crystallographic information

### Comment

The title compound is a kind of ionic liquids to be used as green alternatives to volatile organic solvents inelectrochemical, synthetic and separation processes. For general background, see: (Ding *et al.*, 2007). We herein report the crystal structure of the title compound (I).

In the molecule of (I), (Fig. 1), the bond lengths (Allen *et al.*, 1987) and angles are within normal ranges. The whole molecule has an inversion symmetry located on the ethane group of the main molecule.

In the crystal structure, weak intermolecular C—H···Br interactions (Table 1) link the molecules into chains along the *b* axis (Fig.2), in which they may be effective in the stabilization of the structure.

### Experimental

The ionic liquid compound was prepared following modified literature procedures (Ding *et al.*, 2007). 1-Methylimidazole (8.21 g, 0.1 mol) was mixed with 1,2-dibromoethane (9.38 g, 0.05 mol) in 100 ml of toluene and refluxed for 24 h; the mixture was cooled to room temperature and filtered. The solids were washed several times with ethyl acetate (800 ml) and the white product dried in vacuum (yield:7.3 g, 54.2%). The product was dissolved in the chloroform and the crystals were obtained by evaporating the chloroform slowly at room temperature for about 9 d.

### Refinement

Carbon-bound H atoms were positioned with idealized geometry [aromatic C—H = 0.93 Å, methylene C—H = 0.97 Å and methyl C—H = 0.96 Å] and refined with fixed isotropic displacement parameters [*U*_iso_(H) = 1.5*U*_eq_(*H*)(methyl C) and *U*_iso_(H) = 1.2*U*_eq_ (aromatic and methylene C)] using a riding model.

### Crystal data

**Table:**

C_10_H_16_N_4_^2+^·2Br^−^	*F*(000) = 348
*M**_r_* = 352.07	*D*_x_ = 1.749 Mg m^−^^3^
Monoclinic, *P*2_1_/*c*	Mo *K*α radiation, λ = 0.71073 Å
Hall symbol: -P 2ybc	Cell parameters from 25 reflections
*a* = 8.4750 (17) Å	θ = 9–13°
*b* = 8.9620 (18) Å	µ = 6.05 mm^−^^1^
*c* = 9.2390 (18) Å	*T* = 293 K
β = 107.73 (3)°	Square, white
*V* = 668.4 (3) Å^3^	0.30 × 0.20 × 0.10 mm
*Z* = 2	

### Data collection

**Table:**

Enraf–Nonius CAD-4 diffractometer	862 reflections with *I* > 2σ(*I*)
Radiation source: fine-focus sealed tube	*R*_int_ = 0.021
graphite	θ_max_ = 25.3°, θ_min_ = 2.5°
ω/2θ scans	*h* = 0→10
Absorption correction: ψ scan (North *et al.*, 1968)	*k* = 0→10
*T*_min_ = 0.264, *T*_max_ = 0.583	*l* = −11→10
1296 measured reflections	3 standard reflections every 200 reflections
1212 independent reflections	intensity decay: 1%

### Refinement

**Table:**

Refinement on *F*^2^	Primary atom site location: structure-invariant direct methods
Least-squares matrix: full	Secondary atom site location: difference Fourier map
*R*[*F*^2^ > 2σ(*F*^2^)] = 0.059	Hydrogen site location: inferred from neighbouring sites
*wR*(*F*^2^) = 0.160	H-atom parameters constrained
*S* = 1.01	*w* = 1/[σ^2^(*F*_o_^2^) + (0.1*P*)^2^ + 0.7*P*] where *P* = (*F*_o_^2^ + 2*F*_c_^2^)/3
1212 reflections	(Δ/σ)_max_ < 0.001
73 parameters	Δρ_max_ = 0.80 e Å^−^^3^
0 restraints	Δρ_min_ = −0.85 e Å^−^^3^

### Special details

**Table:**

Geometry. All e.s.d.'s (except the e.s.d. in the dihedral angle between two l.s. planes) are estimated using the full covariance matrix. The cell e.s.d.'s are taken into account individually in the estimation of e.s.d.'s in distances, angles and torsion angles; correlations between e.s.d.'s in cell parameters are only used when they are defined by crystal symmetry. An approximate (isotropic) treatment of cell e.s.d.'s is used for estimating e.s.d.'s involving l.s. planes.
Refinement. Refinement of *F*^2^ against ALL reflections. The weighted *R*-factor *wR* and goodness of fit *S* are based on *F*^2^, conventional *R*-factors *R* are based on *F*, with *F* set to zero for negative *F*^2^. The threshold expression of *F*^2^ > σ(*F*^2^) is used only for calculating *R*-factors(gt) *etc*. and is not relevant to the choice of reflections for refinement. *R*-factors based on *F*^2^ are statistically about twice as large as those based on *F*, and *R*- factors based on ALL data will be even larger.

### Fractional atomic coordinates and isotropic or equivalent isotropic displacement parameters (Å^2^)

**Table:**

	*x*	*y*	*z*	*U*_iso_*/*U*_eq_	
Br	0.71826 (11)	0.14503 (9)	0.46360 (9)	0.0320 (3)	
N1	0.8500 (8)	0.6154 (7)	0.3322 (7)	0.0266 (15)	
C1	0.9914 (11)	0.5837 (12)	0.2817 (11)	0.048 (3)	
H1A	0.9870	0.4818	0.2488	0.072*	
H1B	1.0912	0.5997	0.3639	0.072*	
H1C	0.9904	0.6486	0.1987	0.072*	
N2	0.6231 (8)	0.5865 (7)	0.3878 (7)	0.0224 (14)	
C2	0.7287 (9)	0.5215 (9)	0.3336 (8)	0.0235 (17)	
H2A	0.7213	0.4231	0.3003	0.028*	
C3	0.6774 (10)	0.7310 (9)	0.4270 (9)	0.0276 (19)	
H3A	0.6270	0.8023	0.4713	0.033*	
C4	0.8169 (11)	0.7482 (9)	0.3886 (10)	0.035 (2)	
H4A	0.8796	0.8349	0.3987	0.042*	
C5	0.4817 (10)	0.5171 (10)	0.4162 (9)	0.0265 (18)	
H5A	0.4544	0.4255	0.3581	0.032*	
H5B	0.3869	0.5834	0.3836	0.032*	

### Atomic displacement parameters (Å^2^)

**Table:**

	*U*^11^	*U*^22^	*U*^33^	*U*^12^	*U*^13^	*U*^23^
Br	0.0506 (5)	0.0167 (5)	0.0261 (5)	0.0002 (4)	0.0081 (4)	−0.0024 (4)
N1	0.039 (4)	0.018 (4)	0.024 (3)	−0.007 (3)	0.011 (3)	−0.008 (3)
C1	0.044 (5)	0.068 (7)	0.039 (6)	−0.016 (5)	0.023 (5)	−0.021 (6)
N2	0.034 (4)	0.015 (3)	0.015 (3)	−0.004 (3)	0.003 (3)	0.004 (3)
C2	0.033 (4)	0.017 (4)	0.018 (4)	−0.005 (3)	0.004 (3)	−0.008 (3)
C3	0.045 (5)	0.011 (4)	0.028 (5)	−0.003 (3)	0.013 (4)	0.000 (3)
C4	0.046 (5)	0.018 (4)	0.040 (5)	−0.012 (4)	0.011 (4)	−0.009 (4)
C5	0.029 (4)	0.026 (4)	0.024 (4)	−0.010 (3)	0.007 (3)	0.001 (4)

### Geometric parameters (Å, °)

**Table:**

N1—C2	1.331 (10)	N2—C5	1.443 (9)
N1—C4	1.363 (10)	C2—H2A	0.9300
N1—C1	1.441 (10)	C3—C4	1.343 (12)
C1—H1A	0.9600	C3—H3A	0.9300
C1—H1B	0.9600	C4—H4A	0.9300
C1—H1C	0.9600	C5—C5^i^	1.514 (15)
N2—C2	1.290 (10)	C5—H5A	0.9700
N2—C3	1.386 (10)	C5—H5B	0.9700
			
C2—N1—C4	107.4 (7)	N1—C2—H2A	124.8
C2—N1—C1	126.9 (7)	C4—C3—N2	106.7 (7)
C4—N1—C1	125.8 (7)	C4—C3—H3A	126.7
N1—C1—H1A	109.5	N2—C3—H3A	126.7
N1—C1—H1B	109.5	C3—C4—N1	107.5 (7)
H1A—C1—H1B	109.5	C3—C4—H4A	126.2
N1—C1—H1C	109.5	N1—C4—H4A	126.2
H1A—C1—H1C	109.5	N2—C5—C5^i^	110.5 (8)
H1B—C1—H1C	109.5	N2—C5—H5A	109.6
C2—N2—C3	108.0 (6)	C5^i^—C5—H5A	109.6
C2—N2—C5	126.1 (7)	N2—C5—H5B	109.6
C3—N2—C5	125.7 (7)	C5^i^—C5—H5B	109.6
N2—C2—N1	110.3 (7)	H5A—C5—H5B	108.1
N2—C2—H2A	124.8		
			
C3—N2—C2—N1	−0.9 (8)	N2—C3—C4—N1	−2.0 (9)
C5—N2—C2—N1	−175.6 (7)	C2—N1—C4—C3	1.5 (10)
C4—N1—C2—N2	−0.4 (9)	C1—N1—C4—C3	−178.1 (8)
C1—N1—C2—N2	179.2 (8)	C2—N2—C5—C5^i^	101.3 (10)
C2—N2—C3—C4	1.8 (9)	C3—N2—C5—C5^i^	−72.4 (11)
C5—N2—C3—C4	176.5 (7)		

Symmetry codes: (i) −*x*+1, −*y*+1, −*z*+1.

### Hydrogen-bond geometry (Å, °)

**Table:**

*D*—H···*A*	*D*—H	H···*A*	*D*···*A*	*D*—H···*A*
C2—H2A···Br	0.93	2.92	3.591 (8)	130
C1—H1B···Br^ii^	0.96	2.97	3.738 (8)	138

Symmetry codes: (ii) −*x*+2, −*y*+1, −*z*+1.
